# C‐type lectin receptors in antifungal immunity: Current knowledge and future developments

**DOI:** 10.1111/pim.12951

**Published:** 2022-10-07

**Authors:** Remi Hatinguais, Janet A. Willment, Gordon D. Brown

**Affiliations:** ^1^ MRC Centre for Medical Mycology University of Exeter Exeter UK

**Keywords:** Immunological terms, innate immunity, PAMP, PRR

## Abstract

C‐type lectin receptors (CLRs) constitute a category of innate immune receptors that play an essential role in the antifungal immune response. For over two decades, scientists have uncovered what are the fungal ligands recognized by CLRs and how these receptors initiate the immune response. Such studies have allowed the identification of genetic polymorphisms in genes encoding for CLRs or for proteins involved in the signalisation cascade they trigger. Nevertheless, our understanding of how these receptors functions and the full extent of their function during the antifungal immune response is still at its infancy. In this review, we summarize some of the main findings about CLRs in antifungal immunity and discuss what the future might hold for the field.

## 1 INTRODUCTION

**FIGURE 1:**
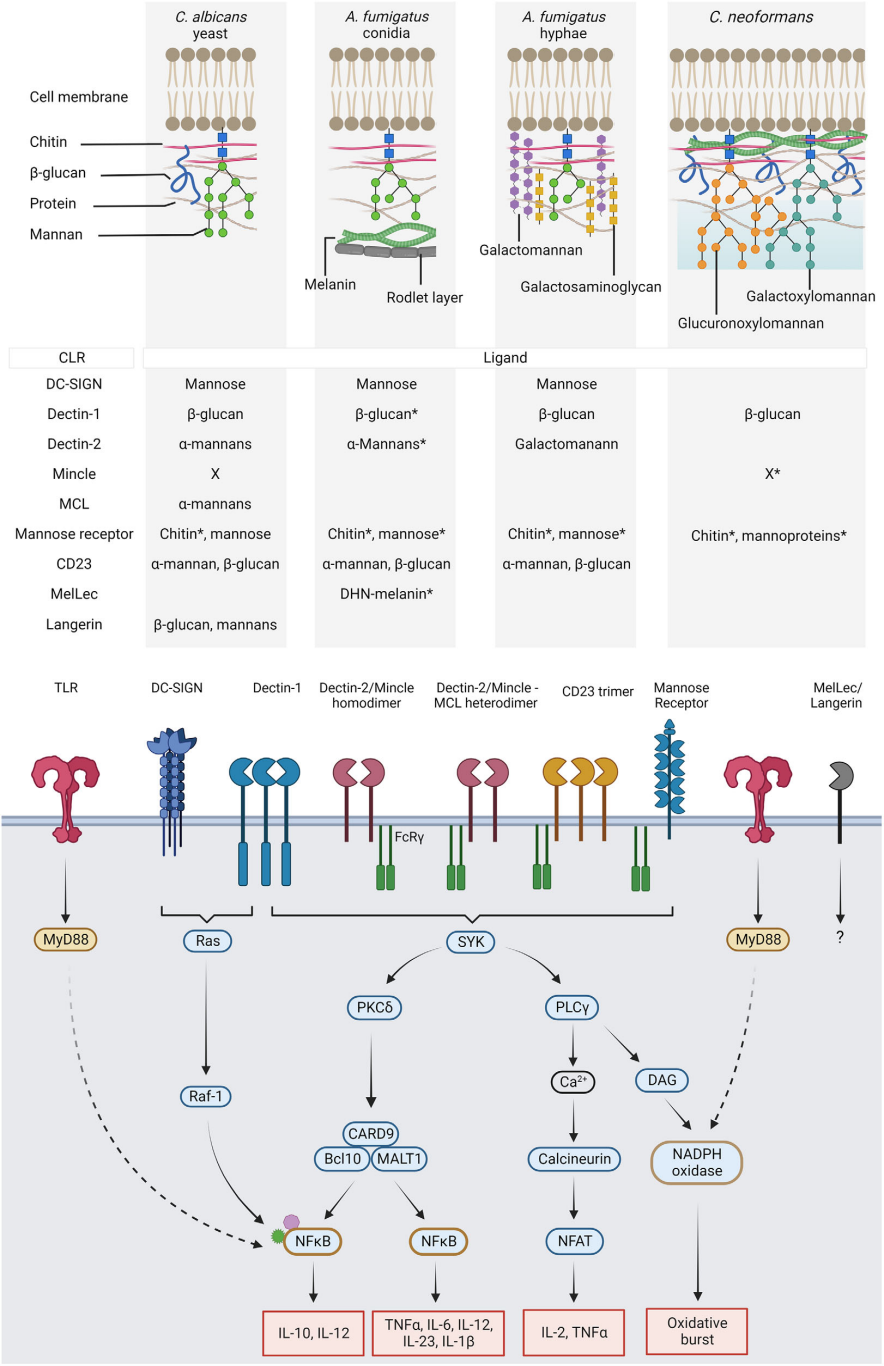
Recognition of the fungal cell wall by C‐type lectin receptors (CLR) initiates the antifungal immune response. *Top*: Simplified representation of the fungal cell wall of the three main fungal pathogens, adapted from Reference [5]. The cell wall of *Candida albicans* (yeast form, left) is composed of chitin, β‐glucans, proteins and mannans. Resting spores of *Aspergillus fumigatus* (centre left) contains chitin, β‐glucan and mannans, which are covered by melanin. The outer layer is composed of proteins from the family of hydrophobins that constitute the rodlet layer, limiting immune recognition of the spores. When conidia germinate into hyphae (centre right), they lose the rodlet layer and the DHN‐melanin but produce galactosaminoglycan and galactomannans. In the cell wall of *Cryptococcus neoformans* (*right*), the melanin is proximal to the cell membrane and is covered by large quantities of glucuronoxylomannans and galactoxylomannans that constitute the capsule (blue shading). *Middle*: Table recapitulating recognition of PAMPs from the above fungal cell wall by CLRs. X indicates that the CLR has been shown to be involved in the recognition of the corresponding fungus but the ligand is not known. *The ligand is present in the cell wall but its accessibility is limited or is growth stage‐dependent. For example, see References [3, 18]. *Bottom*: Collaboration between CLRs and Toll‐Like Receptors (TLRs) induces a robust inflammatory response. CLRs of the Group II can form homodimers or heterodimers with MCL and activate the Spleen Tyrosine Kinase (SYK) pathway by coupling with FcRγ, which is also involved in signal transducing following activation of the mannose receptor (Group VI) or CD23 homotrimers. SYK activation by Dectin‐1 (CLR group V) requires the clustering of Dectin‐1 monomers. SYK activates the phospholipase Cγ (PLCγ) that produces diacylglycerol (DAG), which activates the NADPH oxidase. IP3 production by PLCγ induces the release of intracellular calcium and the translocation of NFAT into the nucleus. SYK also activates the PKCδ, which activates the CARD9‐Bcl10‐MALT1 complex, which in turn activates NFκB. The expression profile induced by NFκB can be modified by post‐translational modifications through the Ras–Raf‐1 pathway that is induced following activation of Dectin‐1 or DC‐SIGN (Group II) tetramers. The production of a robust inflammatory response (red boxes) through the secretion of cytokines and chemokines, as well as the production of the oxidative burst through the activation of NADPH oxidase requires collaboration between the CLR‐SYK (blue boxes) and the TLR‐MyD88 (brown boxes) pathways. The signalling cascades induced by triggering of MelLec and Langerin are not known.

Alongside bacteria, viruses and parasites, fungi constitute a class of pathogens responsible for superficial infections of the mucocutaneous surfaces and for invasive infections with high rates of mortality.^1^ With a few exceptions, invasive infections occur almost exclusively in immunocompromised patients, either due to inheritable genetic mutations, also called primary immunodeficiencies, or secondary to an underlying condition (such as infection by the Human Immunodeficiency Virus) or medical immunosuppression (to prevent graft rejection for instance),^1^, ^2^ Because the majority of invasive infections occur in patients whose immune response is compromised, it is essential to understand the underlying mechanisms behind antifungal immunity if we are to develop novel strategies to combat these diseases. The initial steps of the mammalian immune response, termed the innate response, relies on the detection of pathogenic products called pathogen‐associated molecular patterns (PAMPs) by the host's cells. Innate recognition is mediated by immune receptors known as pattern recognition receptors (PRRs).^3^ Several classes of PRRs have been identified such as toll‐like receptors (TLRs), NOD‐like receptors (NLRs), RIG‐I‐like receptors (RLRs) and C‐type‐lectin receptors (CLRs).^4^ The latter in particular, have been shown to be essential mediators of the antifungal immune response by binding to components of the fungal outer cell wall (Figure 1), and therefore are a direct interface between the fungal pathogens and immune cells.^5^ To date, nine CLRs have been shown to directly bind fungi (Figure 1). In this commentary, we present a brief overview of the role of CLRs in antifungal immunity, highlighting potential future avenues for the field.

### 1.1 C‐type lectin receptors initiate the antifungal immune response

C‐type lectins constitute a superfamily of transmembrane and secreted proteins involved in a variety of biological functions ranging from the conservation of homeostasis to the initiation and modulation of immune responses.^6^ C‐type lectins are divided into 17 subgroups noted from I to XVII, based on their general structure; they contain at least one typical domain: the C‐type lectin‐like domain (CTLD), which confers the ability to bind ligand(s).^7^ The first CLR identified as a PRR for fungi was Dectin‐1 (also called C‐type lectin domain family 7 member A, CLEC7A), a CLR from the subgroup V that recognizes β‐glucan, a carbohydrate ubiquitously present in the cell wall of fungal pathogens.^5^, ^8^ Dectin‐1 belongs to a cluster of CLRs, located on chromosome 12 in human and chromosome 6 in mice, named the ‘Dectin‐1 cluster’ and that include another fungal PRR: the dihydroxynaphtalne (DHN) melanin‐sensing lectin MelLec (CLEC1A).^9^ Most of the CLRs that recognize fungi belong to the Group II subfamily of CLRs. These include dendritic cell‐specific intercellular adhesion ICAM‐3‐Grabbing non‐integrin (human dendritic cell [DC]‐SIGN and its 8 mouse homologues), CD23, Langerin and the members of the ‘Dectin‐2 cluster’ (syntenic to the Dectin‐1 cluster): Dectin‐2 (CLEC6A), Macrophage C‐type Lectin (MCL, CLEC4D), Mincle (CLEC4E) and Langerin.^7^, ^10^ CLRs from the Groups II and VII contain a single CTLD; by contrast, the Mannose Receptor, which belongs to the Group VI and also binds fungi, contains 8–10 CTLDs in its extracellular domain (Figure 1).^7^ Most of these CLRs are expressed by myeloid cells, mainly monocytes, macrophages, DCs and granulocytes.^11^ Unusually, MelLec appears to be primarily expressed by endothelial cells in mice and human, although studies have suggested that this receptor may be expressed on myeloid cells in some species (including humans).^12^, ^13^

Binding of CLRs to their respective ligand initiates a signalling cascade resulting in several cellular responses and the initiation of an inflammatory response, which is key to successfully combating fungal infections. Most myeloid CLRs (such as CD23, the mannose receptor, as well as Dectin‐2 and Mincle, which can form heterodimers with MCL) do not possess a cytoplasmic signalling motif but associate with immunoreceptor tyrosine‐based activation motif (ITAM)‐bearing FcRγ chain, which enables intracellular signalling.^3^, ^14^, ^15^ Following binding of the CLR to its ligand, the ITAM becomes phosphorylated and recruits the Spleen Tyrosine Kinase (SYK). Although Dectin‐1 does not associate with FcRγ, it can still activate SYK due to its incomplete ITAM motif (termed hemITAM) present in its cytoplasmic tail.^3^, ^14^ Dectin‐1‐mediated activation of SYK requires Dectin‐1 monomers to cluster at the cell membrane in contact with its ligand. Once activated, SYK phosphorylates several targets, including the Phosphoinositide‐3‐Kinase (PI3K) and the Phospholipase Cγ (PLCγ).^16^ PLCγ dissociates the phosphatidylinositol (3,4,5)‐triphosphate (PIP3) generated by the phosphoinositide‐3‐kinase into inositide‐3‐phosphate (IP3) and diacylglycerol (DAG).^16^ IP3 increases the intracellular calcium concentration, leading to the translocation of Nuclear Factor of Activated T cells (NFAT) into the nucleus.^3^, ^16^ DAG activates the Protein Kinase C (PKC) and the NADPH oxidase, responsible for the production of Reactive Oxygen Species (ROS) and thereby creating an oxidative burst.^3^, ^16^ PKC also activates the adaptor protein CARD9, which forms a complex with MALT1 and Bcl10 to activate the Nuclear Factor κB (NFκB).^3^, ^16^ Not all CLRs activate SYK following ligand binding. Triggering of DC‐SIGN by mannosylated ligands activates the Ras–Raf1 pathway, which can also be activated by Dectin‐1.^3^, ^14^ Noteworthy, different ligands could induce different signalling cascades by CLRs, for instance, binding of DC‐SIGN by fucosylated ligands induces another signalling cascade resulting in the activation of Bcl3 and promotion of Th2 polarization.^14^ The signalling pathways induced by MelLec and Langerin have not yet been defined. Importantly, triggering of CLRs alone does not induce a robust immune response. Robust responses require a co‐stimulation signal, usually, through the activation of other PRRs.^3^, ^17^ The best‐described signalling partners of CLRs are TLRs, whose signalling primarily involves Myeloid Differentiation factor 88 (MyD88) activation, resulting in the translocation of NFκB into the nucleus (Figure 1).^3^, ^17^ The SYK and MyD88 pathways synergise with each other to induce the secretion of large amounts of pro‐inflammatory cytokines, inducing local inflammation and of chemokines, attracting more leukocytes to the site of infection.^3^, ^17^

In addition to the secretion of inflammatory mediators, CLRs are involved in the internalization of fungi and the initiation of fungicidal programs, which are tightly tailored based on the target's size.^19^, ^20^ If the pathogen can be phagocytosed, the killing of occurs inside the cell, after fusion of the phagosome with lysosomes. Fungal killing is mediated by the production of ROS, antimicrobial proteins and exposure to acidic pH.^20^ If the target is too large to be engulfed, for instance in the case of hyphae, CLRs induce the extracellular release of ROS and antimicrobial proteins.^20^ When encountering *C. albicans* hyphae, neutrophils can release Neutrophil Extracellular Traps (NETs), a ‘net’ consisting of antimicrobial proteins and nuclear DNA, trapping and killing the fungus.^19^, ^21^ Notably, the killing of *A. fumigatus* hyphae appears to rely on the oxidative burst, although large hyphae from this fungus also induce the release of NETs.^20^, ^22^

CLRs also play a role in initiating and polarizing the adaptive phase of the immune response. In antigen‐presenting cells such as DCs, signalling from CLRs promotes antigen presentation, expression of co‐stimulatory molecules such as CD80/CD86 and the secretion of cytokines required for CD4^+^ T helper (Th) polarization.^23^ CLRs are able to drive the adaptive response toward the differentiation of Th1 and Th17 cells, which are considered protective in antifungal immunity.^23^ In some instances, CLRs also promote the expansion of Th2 cells that are thought to have a detrimental role in combatting fungal infections.^23^ Th2 responses also contribute to the development of allergic responses. In fact, fungal sensitisation in allergy is underappreciated, and affects up to 10% of the population and 35%–75% of asthmatic patients, for whom it is associated with higher risk of severe asthma attacks.^24^ Although less well understood, regulatory T cells (Tregs) can limit tissue damage in airway infection models by fungi, and prevent the development of Th2 allergic reactions.^23^ However, in some models, they also play a detrimental role and negatively affect mouse survival following systemic infection.^25^ The adaptive response also encompasses the activation of cytotoxic CD8^+^ T cells and B cells, however, the role of these subsets in antifungal immunity and how CLRs affect those responses is less clear.

The importance of the SYK‐CARD9 pathway triggered by CLRs in antifungal immunity is illustrated by the existence of human *CARD9* mutations, which are associated with significantly increased susceptibility to fungal infections such as systemic candidiasis^26^ and deep dermatophytosis by *Trichophyton* spp.^2^ In some instances, *CARD9* mutations can promote the development of allergic responses to fungi, including allergic broncho‐pulmonary aspergillosis.^27^ Yet, CARD9‐deficient patients do not appear to be more susceptible to infections by bacterial, viral or parasitic pathogens, highlighting the unique role of CLRs in the antifungal immune response.^2^ In contrast to the essential function of CARD9, individual CLRs appear to exhibit a certain level of redundancy in humans and mice. In the absence of immunosuppressive comorbidities, single nucleotide polymorphisms (SNPs) in genes encoding for individual CLRs appear to increase the susceptibility to superficial diseases but not invasive disease. For instance, an SNP in *CLEC7A* (encoding Dectin‐1), was shown to predispose carriers to chronic mucocutaneous colonization by *C. albicans* but not disseminated candidiasis.^28^ Nevertheless, in a context of immunosuppression such as haematopoietic transplantation, the same SNP was shown to increase the risk of invasive aspergillosis, regardless of whether the mutation was carried by the donor or the recipient.^29^

## 2 CLRS IN ANTIFUNGAL IMMUNITY: FUTURE DIRECTIONS

### 2.1 Collaboration between CLRs and other PRRs

As discussed above, CLRs collaborate with other PRRs to mount an efficient immune response, which therefore represents an opportunity for therapeutic manipulation. A direct application of this is illustrated by the case of chromoblastomycosis, a chronic skin infection by the fungal pathogen *Fonsecaea pedrosoi*.^30^ This fungus is recognized by Mincle but not by any TLRs and immune cells consequently fail to initiate a robust immune response, leading to chronic infection.^30^ However, the absence of TLR stimulation by *F. pedrosoi* can be compensated for by the exogenous addition of agonists such as imiquimod, a topical TLR7 ligand.^31^ Restoring the collaboration between TLRs and CLRs was shown to be efficient in patients, in which the use of imiquimod greatly improved the clearance of the pathogen.^32^, ^33^ While the collaboration between CLRs and TLRs has been extensively characterized in the context of antifungal immunity, CLR‐induced potentiation of TLR‐mediated responses is likely to depend on the inflammatory environment. For instance, in a case of liver sterile inflammation, Dectin‐1 dampens rather than potentiates the inflammatory response induced by TLR4, which resulted in reduced liver fibrosis.^34^ Therefore, much remains to be discovered and new findings could constitute therapeutic strategies to modulate CLR‐mediated responses not only during antifungal infection but also in other inflammatory diseases.

How CLRs collaborate with PRRs other than TLRs remains poorly characterized; yet understanding this will probably shed light on underappreciated pathophysiological mechanisms and new potential therapeutics. One of the intracellular PRRs involved in antifungal immunity is the NOD‐ LRR‐ and pyrin‐containing protein 3 (NLRP3), a member of the NLR family. Following binding of β‐glucans by NLRP3, this protein induces the formation of a multiprotein complex called the inflammasome. Once assembled, the NLRP3 inflammasome requires a number of cellular cues (for review see Reference [35]) to become activated. After activation, the inflammasome cleaves zymogens of the IL‐1β family, a potent class of pro‐inflammatory cytokines, into their active form and thereby allows their release.^35^ NLRP3 has been shown to bind directly to fungal products such as β‐glucans and galactosaminogalactan, which induce the assembly of the inflammasome complex.^36^, ^37^ Nevertheless, secretion of IL‐1β during fungal infections involves collaboration between NLRP3 and CLRs since the SYK‐CARD9 pathway is required for the production of pro‐IL‐1β, and for the activation step of the inflammasome following its assembly.^36^, ^38^, ^39^ Activation of the NLRP3 inflammasome is essential for protection against disseminated candidiasis^38^ and for control of airway colonization by *A. fumigatus*.^40^ In spite of this protective effect, the inflammasome is responsible for a number of inflammatory disorders related to fungal infections. The inflammation induced by the cytokines of the IL‐1β family has been associated with symptomatic recurrent vulvovaginal candidiasis,^41^ colitis (as discussed below), and pathological inflammation in cystic fibrosis following *A. fumigatus* challenge.^40^ More studies are needed to fully appreciate the extent to which CLRs collaborate with other PRRs, how this affects the antifungal immune response, and whether targeting CLRs to modulate pathological inflammation could constitute a therapeutic approach.

### 2.2 Regulation of CLR signalling and function

Through the study of CLR signalling, several negative regulators of the SYK cascade have been identified and could constitute therapeutic targets to potentiate the antifungal activity of these PRRs. One of those negative regulators is the ubiquitin ligase Casitas b lymphoma b (Cbl‐b) that is phosphorylated by SYK following triggering of Dectin‐1 or Dectin‐2.^42^ Once activated, Cbl‐b induces the ubiquitination of both these CLRs and SYK, targeting them for proteasome degradation, thereby dampening further activation via these receptors.^42^ In a mouse model of systemic candidiasis, the active Cbl‐b suppressed fungal clearance. Excitingly, pharmacological inhibition of this ubiquitin ligase reduced fungal burdens and greatly improved animal survival, suggesting therapeutic opportunities.^43^ Other negative regulators of CLR signalling have been identified, yet their relevance in vivo is not always clear.^44^, ^45^ Nevertheless, as antifungal drug resistance cases are on the rise,^46^ rewiring the antifungal immune response is an exciting therapeutic avenue that could be used as a replacement or in combination with current antifungals.

Besides intracellular regulators that affect the signalling cascade, the role of CLRs can be modulated by the microenvironment, which could strongly influence the antifungal immune response and susceptibility to fungal infections. Cytokines can modulate the signalling of CLRs by inhibiting the activation of signalling intermediates such as SYK.^47^ Cytokines can also influence the expression of CLRs at the surface of immune cells, which could play an important role when the inflammatory environment changes over time such as during chronic infections or in allergic settings. IL‐4, a Th2‐associated cytokine is known to increase the expression of Dectin‐1, whereas it represses the expression of Dectin‐2, Mincle and MCL.^48^, ^49^ IL‐4 has well‐described effects on the polarization of Th2 cells,^50^ yet how Th2 cytokines modulate CLR expression in vivo and how this might contribute to the maintenance of allergic reactions and tissue remodelling, a major complication of allergy, remains unclear.

Another context in which the inflammatory environment might affect the function of CLRs in the antifungal immunity is the one of co‐infections. A large number of studies have highlighted the high frequency of viral and fungal co‐infections. Influenza is a well‐known risk factor for invasive aspergillosis^51^ and SARS‐CoV2 is associated with aspergillosis^52^ and mucormycosis, a range of fungal infections caused by fungi from the *Mucorales* order.^53^ Importantly, bacteria are frequently present, as high as 24% in the cases of candidemia.^54^ While a number of studies have shown that co‐infections affect the resistance of both pathogens to antimicrobial drugs, their respective metabolisms, and their morphology (such as the ability of *C. albicans* to form hyphae),^55^, ^56^ is still poorly characterized. Furthermore, the impact of these co‐infections on CLR expression and function is unknown.

Lastly, in addition to inflammatory mediators, metabolic cues could also affect CLR‐mediated antifungal responses. Indeed, some authors have recently suggested that glucose availability might affect macrophage responsiveness to cytokines, how this affects CLR expression or function is unknown.^57^ Notably, changes in concentrations of metabolites such as glucose and iron also affect the composition of the fungal cell wall, with direct consequences on immune recognition that also needs to be taken into account.^58^ Local or systemic perturbation of metabolite concentrations could have a strong effect on antifungal immune responses and are likely to underlie some cases of fungal susceptibility such as the predisposition of diabetic patients to superficial fungal infections.^59^

### 2.3 Influence of CLR endogenous ligands on antifungal immune responses

In addition to fungal PAMPs, CLRs can also bind endogenous ligands, which can modulate the immune response to fungi. For example, Dectin‐1 has well‐described functions in regulating neuroinflammation through the recognition of multiple endogenous ligands that can be either secreted or present at the cell surface.^60^ Mincle can bind to ligands released by dead cells such as β‐glucosylceramides and also free cholesterol.^61^, ^62^ To date, the role of these endogenous ligands in antifungal responses is unexplored. They could act as competitors for fungal PAMPs or be responsible for dampening, increasing or modulating the inflammatory response since binding of different ligands by CLRs can induce unique responses.^14^ Interestingly, cholesterol was shown to potentiate the Mincle‐induced inflammatory response induced by other ligands.^62^ How cholesterol affects the Mincle‐mediated inflammation during fungal infection is unknown, even though it could provide an interesting link between diet and fungal infections.

Besides the CLRs mentioned above that directly bind to fungi, a number of CLRs recognize endogenous ligands and could indirectly participate to the antifungal immunity. Through the recognition of endogenous ligands, CLRs play role in numerous biological functions in ensuring a normal development and the maintenance of homeostasis.^6^ During the elimination of dying cells, recognition of F‐actin by DNGR‐1 (CLEC9A, a member of the Dectin‐1 cluster) promotes the activation of naïve cytotoxic CD8^+^ T cells, in a process termed cross‐presentation.^63^ CD8^+^ T cells are required to clear the intracellular fungal pathogen *Histoplasma capsulatum*.^64^ Nevertheless, the role played by CD8^+^ T cells during antifungal immunity is poorly understood. In addition to activating naive cytotoxic T cells, DNGR‐1 contributes to reduce the sterile inflammation induced by dead cells, including in a disseminated *C. albicans* infection model.^65^ However, the presence of endogenous ligands can also be detrimental for the host, for example, binding of cholesterol by Mincle was shown to promote inflammation, tissue fibrosis and the development of allergic reactions in a skin injury model.^66^ Importantly, because endogenous ligands are likely to still be accessible once the fungal pathogen is cleared, their recognition by CLRs could play a considerable role in the fungal‐associated comorbidities such as tissue remodelling. Deciphering the pathophysiological mechanisms underlying those comorbidities could lead to the development of better strategies to prevent the long‐term consequences of fungal infections.

### 2.4 CLRs and microbiota

The importance of the microbiota on homeostasis and immunity has clearly been demonstrated over the last two decades.^67^ Most studies have characterized the bacterial species found at different localisations and their effects on human health, however, fewer studies have focused on the fungal compartment of the microbiota, termed the mycobiota.^68^ The impact of the mycobiota is likely to be profound. Indeed, fungi have been shown to influence a range of pathologies from inflammatory bowel diseases (IBDs) such as colitis and Crohn's disease, to ductal pancreatic cancer.^68^, ^69^ They also contribute to protect from other fungal infections, for instance, Th17 responses to airborne pathogens partly arises from the expansion of memory CD4^+^ T cells induced by the *C. albicans* in the gut.^70^

There are growing evidences for a role in CLRs in controlling the mycobiota composition and mycobiota‐associated pathologies. As mentioned above, an SNP in *CLEC7A* renders carriers more susceptible to colonization of the mucocutaneous surfaces by *C. albicans*, resulting in recurrent and chronic vulvovaginal candidiasis.^28^ Similarly, in the murine gut, deficiency for Dectin‐1 promotes GI tract colonization by *Candida tropicalis*, which aggravates the symptoms of colitis.^71^ Therefore, by controlling the prevalence of specific fungal species, Dectin‐1 exhibits a protective role in preventing the inflammatory symptoms of colitis. Interestingly, in other models of colitis, Dectin‐1 contributes to pathological inflammation by promoting the recruitment of inflammatory macrophages to the gastrointestinal (GI) tract and inflammasome activation,^72^ which highlights the complex role of Dectin‐1 plays in this setting. In addition to controlling mucosal colonization by fungi, CLRs participate in the barrier function of those sites and prevent the dissemination of fungi into the bloodstream. Dectin‐1 and Dectin‐2 have been shown to promote secretion of IgA in the gut and agonists of those receptors could constitute interesting vaccine adjuvants to promote the mucosal adaptive response.^73^ In mice, Mincle contributes to the establishment of a local tonic Th17 response that prevents microbiota from translocating into the circulation.^74^ Although this effect appeared to be mediated through the recognition of bacteria by Mincle, it is tempting to hypothesize that fungi could also drive such response.

More studies are needed to determine what pathologies are influenced by the mycobiota and how those are influenced by CLRs. It is clear that future studies are required to dissect the function of CLRs in colitis and other IBDs, the role of Dectin‐1 and other CLRs, such as MCL or SIGNR3 (the closest mouse homologue of human DC‐SIGN) is not completely understood.^75^, ^76^ Lastly, most of the studies on the mycobiota have focused on the presence of fungi in the GI tract, nevertheless fungi are found on all mucosal surfaces and are likely to have unappreciated impacts on human health. Although the presence of *C. albicans* in the airways was reported decades ago,^77^ its clinical significance was poorly understood, and only recently has been shown to be a major driver of asthma.^78^ Furthermore, the role CLRs play in the airway colonization by *C. albicans* and the development of fungal sensitisation is still unknown. Altogether, it is clear that our understanding of the effect of mycobiota on health is still at its infancy. Understanding how CLRs alter mycobiota composition, contribute to the maintenance of the equilibrium between the mycobiome and its host or participate in disease progression will provide insight into important pathophysiological mechanisms, which are likely to reveal new therapeutic strategies for the treatment of mycobiota‐related conditions.

## 3 CONCLUDING REMARKS

**FIGURE 2:**
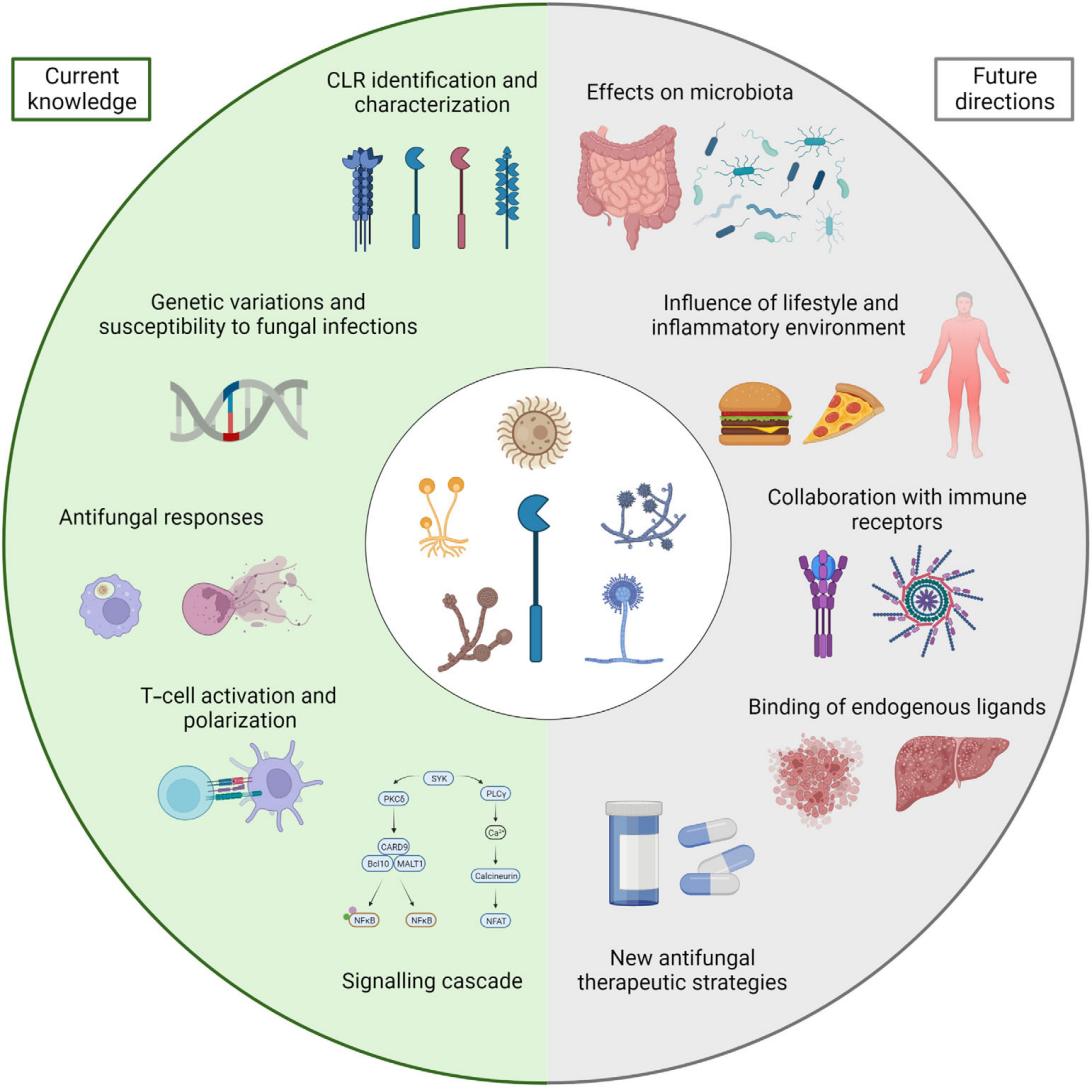
C‐type lectin receptors (CLRs) in antifungal immunity, current knowledge and future developments.

Over the last two decades, CLRs have been identified as key actors of antifungal immunity, initiating innate immune responses and directing the development of adaptive immunity^23^ (Figure 2). The identification of SNPs in the genes encoding for CLRs has allowed us to better understand susceptibility to fungi and are a powerful tool to predict the risk of fungal infections, which could be lifesaving in the case of immunocompromised patients.^12^ Dissecting the signalling of these receptors has highlighted the central function of the adaptor molecule CARD9 in antifungal immunity^26^; it has also, for example, provided us with the immunological mechanisms underlying chronic infections by *Fonsecaea* spp. and how to treat them.^30^ In order to better understand the role of CLRs in antifungal immunity, future studies will have to integrate the function of these receptors with the inflammatory and metabolic environment, and to consider the presence of endogenous ligands that could also contribute to modulation of the immune response. Studies on the impact of the composition of the mycobiota, the role played by CLRs in shaping its composition and mediating its physiological effects have already provided us with exciting results, and it is very likely that more will be revealed in the future. Overall, CLRs still constitute an important reservoir of potential future therapeutic strategies, including by modulating or blocking their signalling.^43^ Other translational approaches include targeting CLRs for the development of vaccines, a missing therapeutic weapon in the management of fungal infections.^6^, ^79^ Excitingly, a Chimeric antigen receptor (CAR) T‐cell therapy using the extracellular domain of Dectin‐1 to provide the CAR‐T cells with a specificity for β‐glucans has shown promising results in treating invasive fungal infections.^80^ Importantly, CLRs, including those primarily described in antifungal immunity, are also involved in the immune response against other pathogenic agents, including parasites, and the antitumoral responses.^6^ Therefore, it is likely that a number of discoveries (past or future) would have significant implications for the management of other pathologies.

The characterization of individual CLRs and their fungal ligands has aided identification of polymorphisms responsible for susceptibility to fungal infections. These studies have enabled us to better understand the mechanisms by which CLRs promote fungal clearance and polarize T cells, notably through deciphering the intracellular signalling cascade induced by CLR triggering.

Future developments in the field, including a better understanding of the interplay between CLRs and the mycobiota, as well as the influence of lifestyle, inflammatory microenvironment, notably with the collaboration of CLRs with other PRRs and cytokine receptors, and the role of endogenous ligand binding during fungal infections will eventually lead to the development of new antifungal therapeutic strategies.

### ACKNOWLDGEMENTS

This research was funded in whole, or in part, by the Wellcome Trust (Grant number 217163 and 102705). For the purpose of open access, the author has applied a CC BY public copyright licence to any author accepted manuscript version arising from this submission. We also thank the Medical Research Council Centre for Medical Mycology at the University of Exeter for funding (MR/N006364/2).

## CONFLICT OF INTEREST

The authors declare no conflict of interest.

### PEER REVIEW

The peer review history for this article is available at https://publons.com/publon/10.1111/pim.12951.

## Data Availability

Data sharing is not applicable to this article as no new data were created or analyzed in this study.
